# Development and comparison of enzyme-linked immunosorbent assays based on recombinant trimeric full-length and truncated spike proteins for detecting antibodies against porcine epidemic diarrhea virus

**DOI:** 10.1186/s12917-019-2171-7

**Published:** 2019-11-27

**Authors:** Chia-Yu Chang, Ju-Yi Peng, Yun-Han Cheng, Yen-Chen Chang, Yen-Tse Wu, Pei-Shiue Tsai, Hue-Ying Chiou, Chian-Ren Jeng, Hui-Wen Chang

**Affiliations:** 1 Graduate Institute of Molecular and Comparative Pathobiology, School of Veterinary Medicine, National Taiwan University, No. 1, Section 4, Roosevelt Rd., Taipei, 10617 Taiwan; 2 School of Veterinary Medicine, National Taiwan University, Taipei, 10617 Taiwan; 3 Graduate Institute of Veterinary Pathobiology, College of Veterinary Medicine, National Chung Hsing University, Taichung, 402 Taiwan

**Keywords:** Porcine epidemic diarrhea (PED), Serology, Indirect ELISA, Spike protein specific ELISA, Human embryonic kidney (HEK)-293 mammalian cell expression system

## Abstract

**Background:**

Since 2010, outbreaks of genotype 2 (G2) porcine epidemic diarrhea virus (PEDV) have caused high mortality in neonatal piglets and have had devastating impacts on the swine industry in many countries. A reliable serological assay for evaluating the PEDV-specific humoral and mucosal immune response is important for disease survey, monitoring the efficacy of immunization, and designing strategies for the prevention and control of PED. Two PEDV spike (S) glycoprotein-based indirect enzyme-linked immunosorbent assays (ELISAs) were developed using G2b PEDV-Pintung 52 (PEDV-PT) trimeric full-length S and truncated S^1–501^ proteins derived from the human embryonic kidney (HEK)-293 cell expression system. The truncated S^1–501^ protein was selected from a superior expressed stable cell line. The sensitivity and specificity of these two ELISAs were compared to immunostaining of G2b PEDV-PT infected cells and to a commercial nucleocapsid (N)-based indirect ELISA kit using a panel of PEDV negative and hyperimmune sera.

**Results:**

The commercial N-based ELISA exhibited a sensitivity of 37%, a specificity of 100%, and a fair agreement (kappa = 0.37) with the immunostaining result. In comparison, the full-length S-based ELISA showed a sensitivity of 97.8%, a specificity of 94%, and an almost perfect agreement (kappa = 0.90) with the immunostaining result. Interestingly, the S^1–501^-based ELISA had even higher sensitivity of 98.9% and specificity of 99.1%, and an almost perfect agreement (kappa = 0.97) with the immunostaining result. A fair agreement (kappa< 0.4) was seen between the commercial N-based ELISA and either of our S-based ELISAs. However, the results of the full-length S-based ELISA shared an almost perfect agreement (kappa = 0.92) with that of S^1–501^-based ELISA.

**Conclusions:**

Both full-length S-based and S^1–501^-based ELISAs exhibit high sensitivity and high specificity for detecting antibodies against PEDVs. Considering the high protein yield and cost-effectiveness, the S^1–501^-based ELISA could be used as a reliable, sensitive, specific, and economic serological test for PEDV.

## Background

Porcine epidemic diarrhea (PED) is a highly contagious disease that causes watery diarrhea, vomiting, electrolyte imbalance, and dehydration in piglets [1]. The morbidity and mortality rates are highly correlated with the age and the immune status of susceptible piglets [2]. The causative agent of PED is a single stranded, enveloped RNA virus named porcine epidemic diarrhea virus (PEDV), which belongs to the genera *Alphacoronavirus*, the family *Coronaviridae*, and the order *Nidovirales* [3]. The PEDV has an approximately 28-kilobase pair genome, including seven open reading frames, and encodes both non-structural and structural proteins [4]. The four major structural proteins are envelope (E), membrane (M), spike (S), and nucleocapsid (N) [1, 2]. The N protein produced during the early infection stages is the most abundant protein throughout the entire viral propagation process [5, 6]. The M protein is anchored on the envelope of the virion, which is formed by a small amount of E protein [7], whereas the S protein protrudes in homologous trimer and shapes crown-like projections on the viral surface [8]. Among these structural proteins, the S protein, a superficial glycoprotein, is responsible for establishing infection and inducing neutralizing antibodies [9, 10]. The S protein can be divided into the S1 region (amino acid [aa] 1–789) and S2 region (aa 790–1383) [11]. Generally, the S1 region, which can be further subdivided into five structural domains (S1^0^, S1^A^, S1^B^, S1^C^, and S1^D^), dominates the viral-host recognition and receptor binding, whereas the S2 region triggers viral fusion and internalization [9, 12]. Additionally, three neutralizing epitopes on the spike protein of PEDV have been reported: including CO-26 K equivalent epitope (COE epitope; aa 499–638) [13], S1D epitope (aa 636–789) [14], and 2C10 epitope (aa 1368–1374) [15]. Recently, the S1^0^, S1^A^, and S1^B^ domains, as well as the C-terminus of S2, were demonstrated to participate in virus neutralization [8, 16, 17]. The functions and the importance of the S protein of PEDV make it a key target for vaccine development and immune status evaluation.

PED was first reported in Europe and Asia in the early 1970s and over the following thirty years, reports emerged from several more countries [18]. Recently, genotype 2 (G2) PEDV, which has a high virulence, was found to affect Asia and America, resulting in significant economic losses in the swine industries, especially in China, Korea, Japan, Taiwan, US, Canada, and Mexico [1, 3]. The mortality rate in seronegative neonatal piglets after high virulent PED infections reached 90–95% [19–21]. Currently, the disease is still circulating in several countries, therefore, evaluating the PEDV-specific immune response of piglets and sows is essential. This evaluation will allow us to determine the efficacy of immunization and to predict the trend of immune status in the field to create strategies for the prevention and control of PED.

Currently, several enzyme-linked immunosorbent assays (ELISAs) have been established and commercialized to detect systemic IgG or mucosal IgA against PEDV [22–27]. Commercially available ELISA kits for PEDV are mainly based on the N protein of PEDV, because N protein is the dominant protein. It is massively produced during the infection procedure and is able to provoke strong immune responses [24]. However, previous studies have revealed that whole virus, N protein, or M protein-based ELISA may detect cross-reactive antibodies against other swine coronaviruses [25]. On the other hand, several PEDV S-based ELISAs have been developed [26–28] and confirmed no cross-reactivity with other porcine coronaviruses [26, 28]. These results suggest that S-based ELISA may be more suitable for detecting PEDV-specific antibodies.

Mammalian expression systems have the unique ability to produce proteins with complex conformational structures containing the appropriate post-translational modifications [29, 30]. The precise folding and appropriate modifications of the target antigens are key for both vaccine and serological assay development. To date, only one G2b S1 (aa 1–781)-based ELISA has been developed from a mammalian protein expression system [26]. We earlier applied human embryonic kidney (HEK) 293 cell derived PEDV-Pintung 52 (PT) trimeric full-length S protein-based ELISA for the detection of antibodies against homologous strains [31, 32]. However, the differences in performance between the trimeric full-length S-based PEDV ELISA, that contains all epitopes spanning the complete viral S protein, and the truncated S-based PEDV ELISA, that contains partial epitopes of the S protein, in the detection of antibodies against heterologous and historic PEDV strains is unknown.

In addition to sensitivity and specificity, cost effectiveness is also an important concern in the development of ELISA kits. In the present study, we first determined the expression level of a panel of different truncated S proteins generated in our previous study [17]. A truncated S protein that comprised the residues of aa 1–501 of the S protein (S^1–501^) was selected as it showed the highest protein yield. The sensitivity, specificity, and interrater agreements of G2b PEDV trimeric full-length S and S^1–501^ protein indirect ELISAs were compared with those of a commercial N-based indirect ELISA kit using a panel of PEDV hyperimmune serums targeting homologous or heterologous strains of PEDVs.

## Results

### Comparison of the protein yield of different truncated PEDV S proteins

In order to select the truncated S protein with the highest expression levels in HEK 293 cells from which to develop a cost-effective ELISA kit, the yield of different truncated PEDV S proteins was compared. The total amount of full-length S protein purified from 40 mL of culture supernatant was 80 μg, compared to the total amount of purified S^1–435^, S^1–485^, S^1–501^, S^1–509^, S^1–575^, and S^1–639^ proteins, which was 113 μg, 1071 μg, 1600 μg, 486 μg, 45 μg, and 422 μg, respectively. Along with the high-purity full-length S protein [32], the S^1–501^ protein was also chosen as a candidate for the development of ELISA due to its superior protein expression level compared to other truncated S proteins in HEK-293 cells. The purity and quality of the S^1–501^ protein were confirmed by sodium dodecyl sulfate (SDS)-polyacrylamide gel electrophoresis (PAGE) (Additional file 1: Figure S1).

### Immunostaining of serum and colostrum samples against G2b PEDV-PT infected Vero cells

**Fig. 1:**
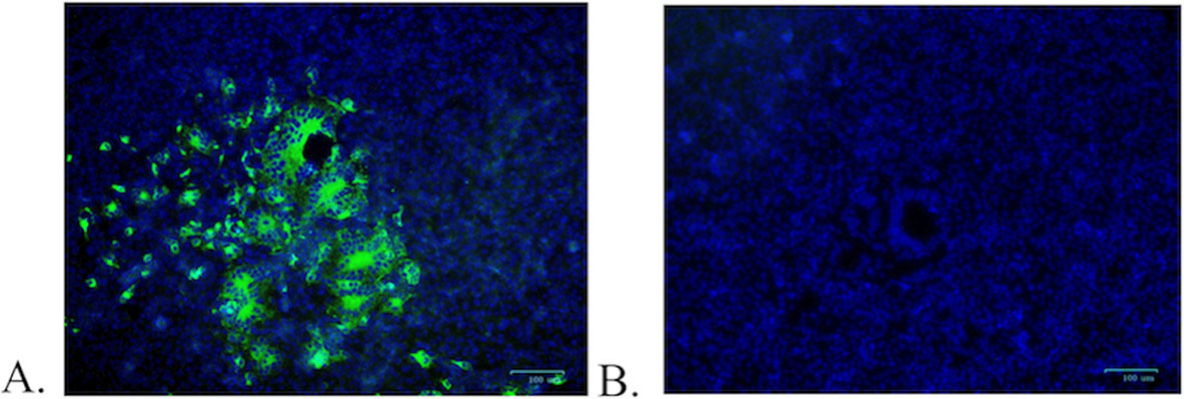
Representative images of immunostaining of serum samples against G2b PEDV-PT infected Vero cells. Vero cells were seeded onto 96-well plates and grown overnight until reaching 90% confluency. The Vero cells were challenged with 1000 TCID_50_/mL of G2b PEDV-PT-P6 diluted in post-inoculation (PI) medium. Vero cells with syncytial cells, the cytopathic effect (CPE) of PEDV, were fixed with acetone after 24 h of incubation. Each serum sample from different sources was serially diluted from 160-fold to 20,480-fold, then added to the plates and incubated for 1 h. Anti-pig IgG antibodies conjugated with horseradish peroxidase (HRP) or fluorescence were used to probe serum IgG on the syncytial cells. A positive signal was found in the Vero cells showing CPE. **a** The representative “positive” result of immunostaining using the serum sample from the C-I group at a 640-fold dilution. **b** The representative “negative” result of immunostaining using the serum sample from the C-N group at a 640-fold dilution. The bars represent the scale of 100 μm

A panel of 213 PEDV negative serum samples and was selected from well-managed, no PEDV exposure history, conventional and SPF farms. A panel of 90 positive serum samples was selected from conventional SPF pigs with PEDV infection history either by experimental G2b PEDV-PT inoculation or by oral feedback using the intestinal homogenate derived from G2b PEDV-infected piglets. The anti-PEDV titers of these serum samples were characterized by either immunofluorescence assay (IFA) or immunocytochemical staining (ICC). The representative immunostained images were shown in Fig. 1. While all samples exhibited high staining backgrounds at the dilutions < 1:160 in the immunostainings, the titer of the serum samples above 1:160 dilution was determined by two-fold serial dilution in our study. The immunostaining titers of all the negative samples, including C-N pigs and SPF-N pigs, were < 1:160, thus confirming them as negative serum samples. The immunostaining titers of the positive group C-I pigs ranged from 1:320–1:5120, including 7 serum samples of 1:320; 8 serum sample of 1:640, 9 serum samples of 1:1280, 15 serum samples of 1:2560, and 11 serum samples of 1:5120. The immunostaining titers of all fifteen SPF-I pigs were consistently 1:5120. The serum titers of the pigs varied from 1:1280–≥1:20480, including 1 serum sample of 1:1280, 5 serum samples of 1:2560, 4 serum samples of 1:5120, 11 serum samples of 1:10240, and 5 serum samples of ≥1:20480. All serum samples from the positive group were confirmed positive by the immunostaining. All colostrum (5/5) of the SPF sows had the titer < 1:160, representing no detectable PEDV-specific IgA. However, all colostrum samples of the conventional sows were positive, including 9 colostrum of 1:160, 6 colostrum of 1:320, 3 colostrum of 1:640, 2 colostrum of 1:1280, and 3 colostrum of 1:2560.

### Determination of assay specificity of commercialized N protein-based ELISA

**Fig. 2:**
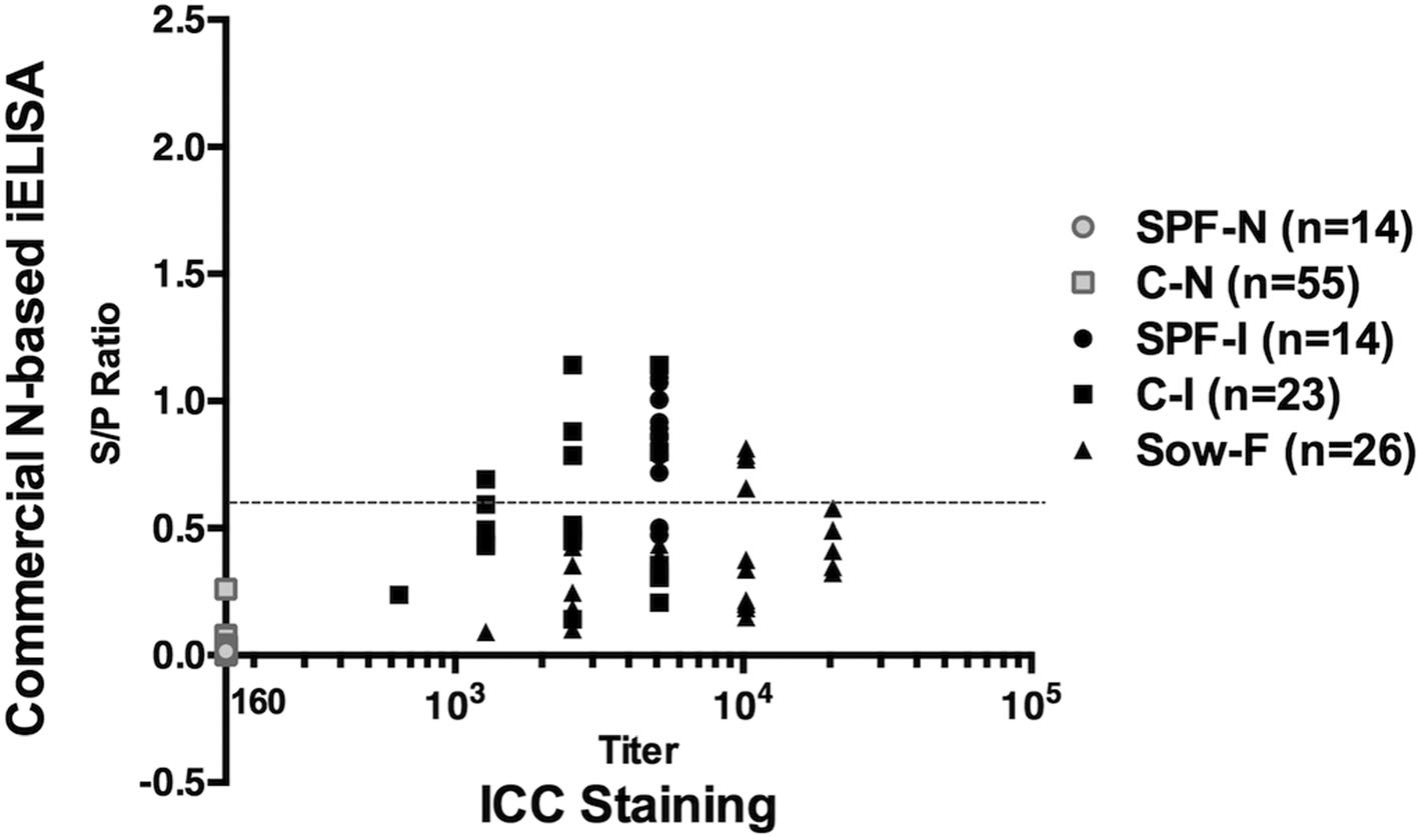
Distribution of the results of the immunostaining and commercial N-based ELISA. The immunostaining titers of each serum sample were presented as logarithms with the base 10. Serum samples which were negative for the immunostaining are denoted by 0. The results of the commercial N-based ELISA kit were presented by the sample to positive ratio (S/P ratio). S/P ratio = [(sample OD – mean OD of negative controls) / (mean OD of positive controls – mean OD of negative controls)]. The dotted line represents the threshold of an S/P ratio of 0.6 of the commercial N-based ELISA, as per the manufacturer’s instructions. C-N (with grey square icon): conventional PEDV naïve pigs; C-I (with black square icon): experimental PEDV inoculated conventional pigs; SPF-N (with grey round icon): specific pathogen free PEDV naïve pigs; SPF-I (with black round icon): experimental PEDV inoculated SPF pigs; Sow-F (with black triangle icon): PEDV-feedback sows

According the manufacturer’s guidelines, the cut-off value of N protein-based ID Screen® PEDV Indirect (IDVet) is 0.6, so the sample to positive ratio (S/P ratio) of the serum sample must be > 0.6 to be considered positive. As shown in Table 1, 55 serum samples randomly picked from the C-N pigs, as well as 14 serum samples from the SPF-N pigs, were found to be negative by screening with the N protein-based ID Screen® PEDV Indirect (IDVet). Twenty-three serum samples were randomly chosen from C-I pigs, eight out of twenty-three (8/23) were determined to be positive, and 15 serum samples were negative. Eleven of fourteen (11/14) serum samples from SPF-I pigs were positive, and only three were negative. On the other hand, four out of twenty-six (4/26) serum samples from Sow-F pigs were positive, and the remaining twenty-two serum (22/26) samples were negative. Using the results of the immunostainings as the gold standard, the sensitivity and specificity of the N protein-based ID Screen® PEDV Indirect (IDVet) were 37 and 100%, respectively. The kappa value between ICC staining and the N protein-based ID Screen® PEDV Indirect (IDVet) was 0.37, which represents a “fair agreement”. The distribution between the results of the N protein-based ELISA and the immunostaining titers is presented in Fig. 2.

**Table 1:** The summary of the result of serological assays in each group. (+): positive; (−): negative; NE: non-examination; C-N: conventional PEDV naïve pigs; C-I: experimental PEDV inoculated conventional pigs; SPF-N: specific pathogen free PEDV naïve pigs; SPF-I: experimental PEDV inoculated SPF pigs; Sow-F: PEDV-feedback sows. NE: non-examined

Group	No. of pigs	Commercial N-based ELISA		Full-length S-based ELISA		S^1–501^-based ELISA	
		+	–	+	–	+	–
C-N	55	0	55	5	50	0	55
	144	NE	NE	6	138	2	142
C-I	23	8	15	23	0	23	0
	27	NE	NE	25	2	26	1
SPF-N	14	0	14	0	14	0	14
SPF-I	14	11	3	14	0	14	0
Sow-F	26	4	22	26	0	26	0
Sensitivity		37%		97.8%		98.9%	
Specificity		100%		94%		99.1%	
Kappa value		0.37		0.90		0.97

### Determination of the cut-off value, sensitivity, and specificity of full-length S-based ELISA

**Fig. 3:**
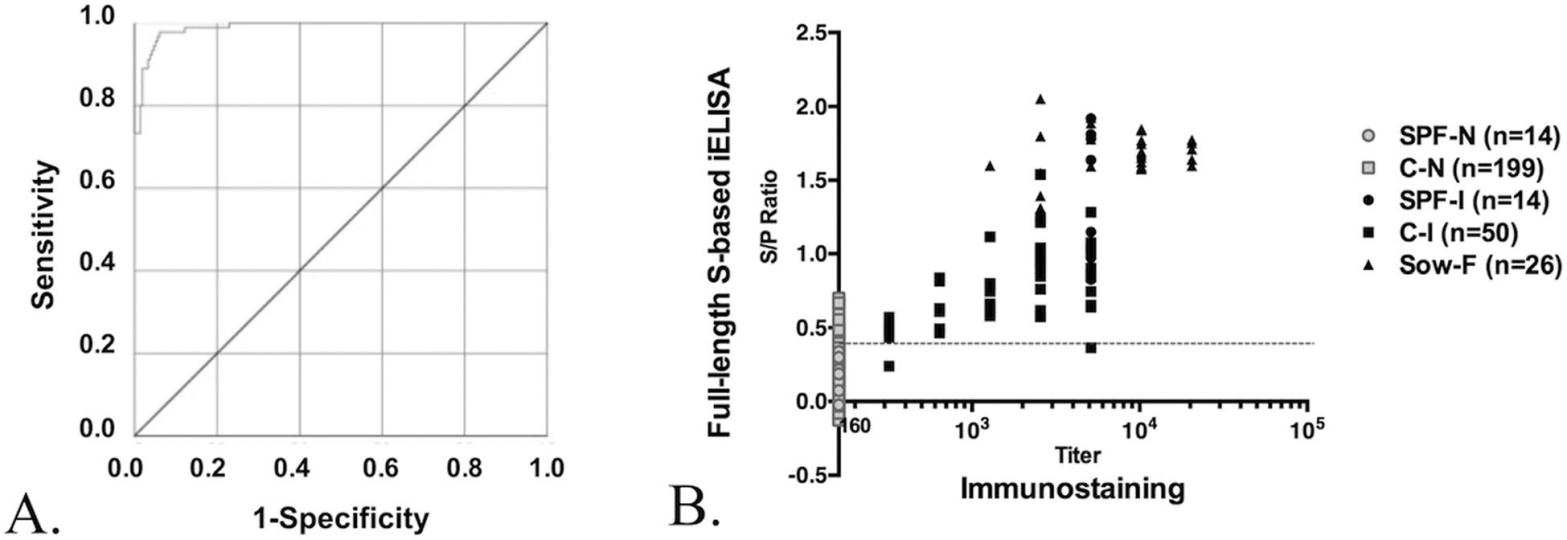
Receiver operating characteristic (ROC) analysis and distribution of the results of the immunostaining and full-length S-based ELISA. **a** The ROC analysis of the full-length S-based ELISA. The sensitivity and specificity were calculated using negative (*n* = 213) and positive (*n* = 90) serum samples. The x axis shows 1-specificity and the y axis shows sensitivity. **b** The immunostaining titers of immunofluorescence assay of each serum sample are presented as a logarithm with base 10. Serum samples that were negative for the immunostaining are denoted by 0. The results of the full-length S-based ELISA are presented as the S/P ratio. The dotted line represents the cut-off value of an S/P ratio of 0.432 as determined by the ROC analysis. C-N (with grey square icon): conventional PEDV naïve pigs; C-I (with black square icon): experimental PEDV inoculated conventional pigs; SPF-N (with grey round icon): specific pathogen free PEDV naïve pigs; SPF-I (with black round icon): experimental PEDV inoculated SPF pigs; Sow-F (with black triangle icon): PEDV-feedback sows

**Fig. 4:**
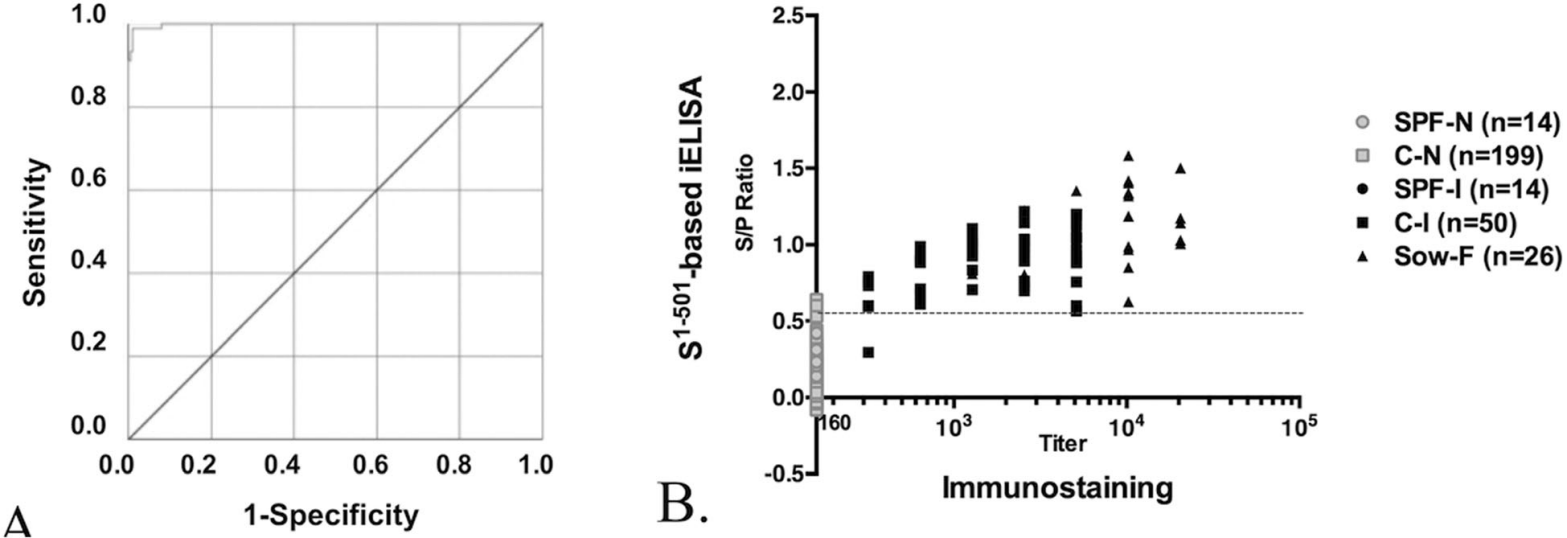
ROC analysis and the distribution of the results of the immunostaining and S^1–501^-based ELISA. **a** ROC analysis of the S^1–501^-based ELISA. The sensitivity and specificity were calculated using negative (*n* = 213) and positive (*n* = 90) serum samples. The x axis shows 1-specificity and the y axis shows sensitivity. **b** The immunostaining titers of immunofluorescence assay of each serum sample are presented as a logarithm with base 10. The serum samples that were negative for the immunostaining are denoted by 0. The results of the S^1–501^-based ELISA are presented as the S/P ratio. The dotted line represents the cut-off value of an S/P ratio of 0.573 as determined by the ROC analysis. C-N (with grey square icon): conventional PEDV naïve pigs; C-I (with black square icon): experimental PEDV inoculated conventional pigs; SPF-N (with grey round icon): specific pathogen free PEDV naïve pigs; SPF-I (with black round icon): experimental PEDV inoculated SPF pigs; Sow-F (with black triangle icon): PEDV-feedback sows

Using the receiver operating characteristic (ROC) analysis as shown in Fig. 4a, the cut-off value of full-length S-based ELISA was determined to be 0.432 to obtain the 97.8% sensitivity and 94% specificity. As summarized in Table 1, starting with a cut-off value of 0.432, forty-eight out of fifty (48/50) serum samples from C-I pigs, fourteen (14/14) serum samples from SPF-I pigs, and twenty-six (26/26) serum samples from Sow-F pigs were found to be positive. Only two out of fifty (2/50) serum samples from C-I pigs had false negative results. Eleven of one hundred ninety-nine (11/199) serum samples from the C-N group had false positive results, while the remaining serum samples (188/199) in the C-N group and all (14/14) serum samples in the SPF-N group were negative. With the kappa value of 0.90, the interrater agreement of the full-length S-based ELISA and the immunostainings is almost perfect. The distribution between the immunostaining titers and the results of full-length S-based ELISA, the distribution is shown in Fig. 3b.

### Determination of cut-off value, sensitivity, and specificity of S^1–501^-based ELISA

**Fig. 5:**
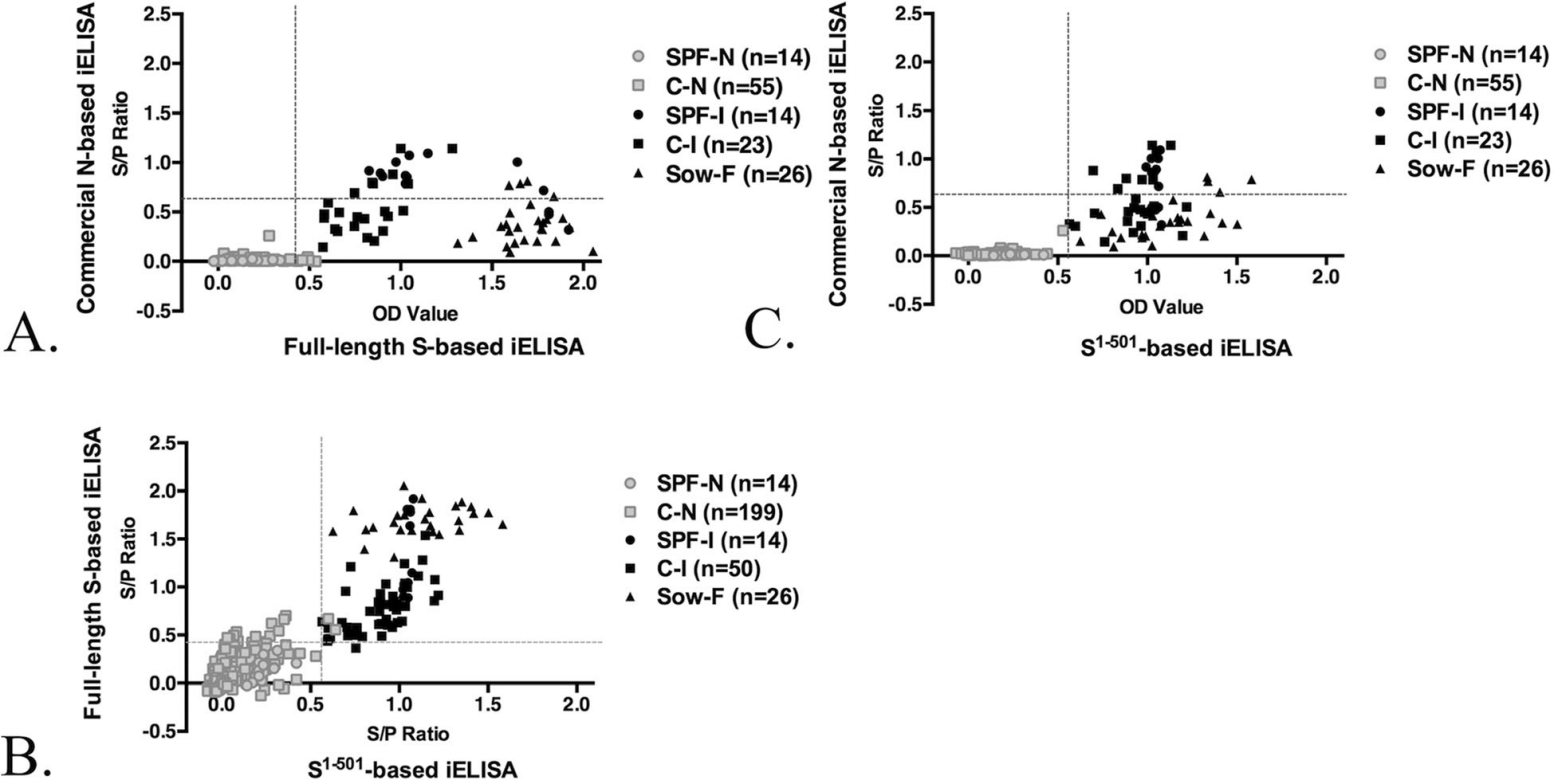
Comparison between commercial N-based, full-length S-based, and S^1–501^-based ELISAs. **a** The distribution of the results of the full-length S-based and commercial N-based ELISAs. The vertical dotted line represents the cut-off value of the full-length-based ELISA. The horizontal dotted line represents the threshold of the commercial N-based ELISAs, as suggested by the manufacturer. **b** Distribution of the results of S^1–501^-based and full-length S-based ELISAs. The vertical dotted line represents the cut-off value of the S^1–501^-based ELISA. The horizontal line represents the cut-off value of the S/P ratio of the full-length-based ELISA. **c** The distribution of the results of S^1–501^-based and commercial N-based ELISAs. The vertical dotted line represents the cut-off value of the S^1–501^-based ELISA. The horizontal dotted line represents the threshold of the commercial N-based ELISAs, as suggested by the manufacturer. C-N (with grey square icon): conventional PEDV naïve pigs; C-I (with black square icon): experimental PEDV inoculated conventional pigs; SPF-N (with grey round icon): specific pathogen free PEDV naïve pigs; SPF-I (with black round icon): experimental PEDV inoculated SPF pigs; Sow-F (with black triangle icon): PEDV-feedback sows

Using the ROC analysis, the cut-off value of S^1–501^-based ELISA was determined to be 0.547 to obtain the 98.9% sensitivity and 99.1% specificity (Fig. 5a). As shown in Table 1 and Fig. 4, except for one serum from the C-I pigs, all C-I (49/50), SPF-I (14/14), and Sow-F (26/26) samples were positive with a cut-off value of 0.547. Except two serum samples (2/199) in the C-N group that were positive, the other serum samples (197/199) in the C-N group and all serum samples in the SPF-N (14/14) group were negative. The kappa value of S^1–501^**-**based ELISA with a cut-off value of 0.547 was 0.97, meaning the results of S^1–501^**-**based ELISA had an almost perfect agreement with that in the immunostaining results (Fig. 4b).

### Agreement comparisons between serological assays

Figure 5a and b showed the distribution of the results between assays. The kappa values between the commercialized N protein-based ELISA and the full-length S-based ELISA and S^1–501^-based ELISA were 0.33 and 0.38, respectively, meaning the assays show fair agreements. In contrast, the agreement between the full-length S-based ELISA and the S^1–501^-based ELISA was almost perfect with a kappa value of 0.92.

### Colostrum IgA detection using full-length S-based and S^1–501^-based ELISAs

**Fig. 6:**
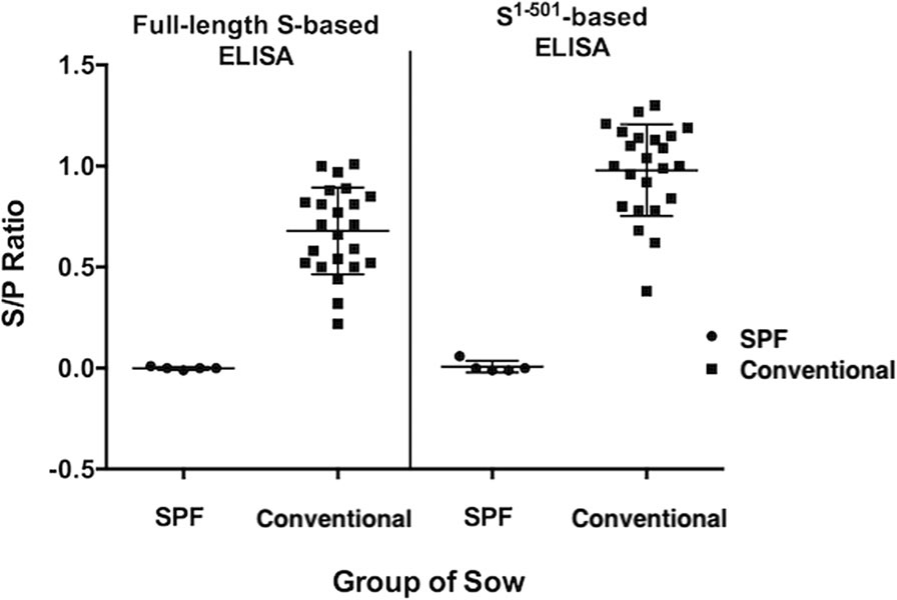
Detection of PEDV S-specific IgA from colostrum using the full-length S-based and S^1–501^-based ELISAs. Colostrum samples from five seronegative SPF sows (*n* = 5, SPF group) and twenty-three feedback conventional sows (*n* = 23, Conventional group) were included. The PEDV S-specific IgA was detected from the colostrum using either full-length S-based and S^1–501^-based ELISAs. The results are presented as the S/P ratio. The round icon represents the result of SPF group; the square icon represents the result of Conventional group. The error bars indicate the mean S/P ratio ± SD

To determine the performance of our S-based ELISAs in detection of colostrum PEDV specific IgA, twenty-three colostrum samples were collected from PEDV sero-positive conventional sows with PED outbreak and feedback history in 2 months as positive control and five colostrum samples were collected from PEDV sero-negative SPF sows as negative control. The PEDV specific IgA titers were determined by using the ICC assay and ranged from 1:160–1:2560 and < 1:160 in positive and negative colostrum samples, respectively. As shown in Fig. 6, the S/P ratio of the colostrum IgA from the PEDV sero-negative SPF sows was approximately 0 ± 0.01 in the full-length S-based or S^1–501^-based ELISA. Importantly, the S/P ratio of the colostrum IgA from the feedback conventional sows, which ranged from 0.22–1.00 and 0.38–1.27 in the full-length S-based and S^1–501^-based ELISA, respectively, was higher than the S/P ratio from PEDV sero-negative SPF sows.

### Characterization and production of S protein of G1b PEDV

**Fig. 7:**
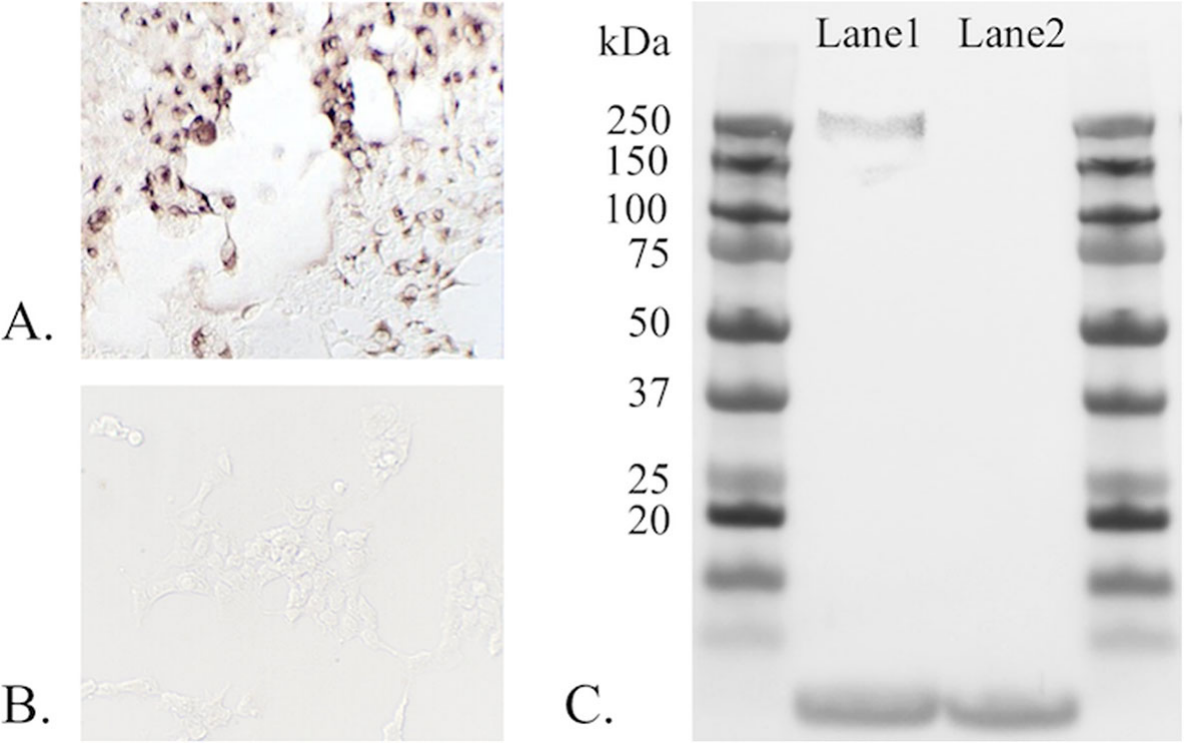
Expression and characterization of G1b PEDV S protein by ICC staining and western blotting. **a** ICC staining of G1b PEDV S-expressing HEK cells using anti-V5 tag antibody. The cells were fixed on the plates using acetone, probed using 1000-fold diluted anti-V5 tag antibody, and detected using anti-mouse IgG antibody conjugated with HRP. The coloration procedure was carried out using the DAB system. **b** The negative control of the ICC staining of non-transfected HEK cells. The cells were probed with anti-V5 tag antibody following by the anti-mouse IgG antibody conjugated with HRP to distinguish the non-specific signals. **c** The molecular weight of the purified G1b PEDV S protein (Lane 1) was estimated by western blotting. The purified G1b PEDV S protein was denatured in NuPAGE® LDS sample buffer containing the NuPAGE® reducing agent and boiled at 95 °C for 5 min, then, separated by SDS-PAGE, transferred onto the PVDF membrane, and stained with anti-V5 tag antibody. The HEK cell lysate was used as the negative control (Lane 2). The ladder is shown in kilodalton (kDa)

We confirmed that both full-length S and S^1–501^-based ELISAs could be used to detect antibodies against homologous or G2b PEDVs. To evaluate whether full-length S and S^1–501^-based ELISAs were able to detect antibodies targeting for heterologous historic PEDVs, the full-length sequence of the S gene of a Taiwan historic G1b PEDV-HC070225 strain was synthesized and the codon optimized for HEK-293 cells. The S protein of the G1b PEDV-HC070225 strain was successfully expressed and characterized via immunocytochemical (ICC) staining with anti-V5 tag antibody (Fig. 7a, b). After protein purification, the molecular weight of the G1bb PEDV S protein was identified at approximately 250 kDa by western blotting (Fig. 7c).

### Evaluation of cross reactivity of serological assays using G1b PEDV S-hyperimmune serum

Following three rounds of immunization, the immunostaining titer of the serum derived from G1b PEDV-HC070225 S protein-immunized pigs was determined to be 1:5120. The S/P ratios of the serum against full-length S-based ELISA and the S^1–501^-based ELISA were 1.58 and 1.17, respectively. Since the cut-off values of S-based ELISA and S^1–501^-based ELISA were 0.432 and 0.573, respectively, the results were interpreted as positive.

## Discussion

Two PEDV S protein-based ELISAs, the full-length S-based and the truncated S^1–501^-based ELISAs, were developed in this study. The standard that was used for comparison in this study is immunostaining against G2b PEDV-PT infected Vero cells. Based on this, our results show that the performance of the S^1–501^-based ELISA in detection of PEDV specific IgG is better than that of the full-length S-based ELISA and the commercialized PEDV N-based ELISA. Furthermore, we also demonstrated that both PEDV S protein-based ELISAs are able to detect the IgA antibodies in colostrum and both G1b and G2 PEDV specific serum IgG. Because of its excellent performance and high protein yield, the S^1–501^-based ELISA is the most affordable and efficient choice for developing serological assays for PEDV.

Several ELISAs for detecting PEDV-specific antibodies have been established in previous studies in which the majority of the coating antigens, including S1, N, or M protein [23, 24, 28, 33], were expressed by a prokaryotic protein expression system to achieve a high yield of protein production. However, protein expressed using a prokaryotic expression system may lack the appropriate protein folding and the complexity of post-translational modifications, such as glycosylation, which could lead to a reduction in the sensitivity and specificity of ELISA kits. In this study, a HEK-293 mammalian expression system was used to express the coating antigens, S proteins, for the development of PEDV ELISAs. The S protein of PEDV is a key target for virus neutralization and vaccine development [1, 4] and contains several neutralizing epitopes [8, 13, 15–17], which may be an important indicator for evaluating immunity against PEDV [1]. The S protein of coronavirus has been demonstrated to display a complex structure and contains at least 21–35 predicted N-glycosylation sites. The mammalian protein expression system allows for proper formation of complex multidimensional structures and post-translational modifications, thereby generating antigens that can attain a high sensitivity in ELISAs [12, 34, 35]. The S protein of PEDV is a trimeric protein [36] and, similar to other coronaviruses, the trimeric form of the S protein may contain more conformational epitopes than a monomeric protein [37]. Therefore, the full length of the trimeric spike protein containing all S1 and S2 epitopes is considered an important target for the development of ELISAs [8, 13–16]. However, the large molecular size of the full-length S protein presents a challenge for large scale protein expression and production [32]. With these difficulties, many studies have preferred to use S1 instead of full-length S to conduct both vaccine and serological assay development [28, 31, 38]. The high sensitivity and specificity of the ELISA developed using the S1 protein of PEDV produced by Freestyle 293-F cells [26] suggests that truncated S proteins might also be good candidates for developing PEDV S-based ELISA. However, the performance of the S1-based ELISA has never been compared with the full-length PEDV S-based ELISA. Based on the comparison of the protein yields of different truncated S protein-expressing stable HEK-293 cells, the S^1–501^-based ELISA was successfully developed. Interestingly, the full-length S-based ELISA was not superior in terms of its ability to detect PEDV-specific antibodies compared with the truncated S^1–501^-based ELISAs. In the present study, the S^1–501^ protein was prepared from the supernatant of a stable cell line to reduce the expenses for transfection and expression. Furthermore, the S^1–501^ expressing stable cell line was selected based on its superior protein expression quantity (40 μg/mL culture supernatant) to others. For ELISA application, approximately 20 μg of S protein was used for preparing a 96-well plate ELISA. These suggest that each mL of culture supernatant could be used to prepare two ELISA plates. The excellent performance of the truncated S^1–501^-based ELISA with the highest protein expression level indicates this would be a good antigen and for PEDV ELISA. Our findings are similar to those of previous reports in which S protein was found to be an ideal antigen for developing ELISA, with a higher sensitivity and better specificity than N protein-based ELISAs [27, 39]. Both full-length S-based and S^1–501^-based ELISAs have much better sensitivities than the commercial N-based ELISA kit. The S-based ELISAs used in this study can be used in both SPF pigs, conventional pigs, and sows, whereas commercial N-based ELISAs only perform well in SPF pigs, which have a relatively low background and stronger immune response. The S-based ELISAs can be used to detect the immune response and the immune status of the population and is an indispensable tool in evaluating the efficacy of PEDV vaccines [39].

Currently, several G1b and G2b PEDV strains are circulating in North America and Asia [40–42]. Therefore, establishing a serological assay that is able to detect anti-G1 and G2b PEDV antibodies is important in order to evaluate the immune status of swine populations. Since there is no clinically confirmed serum against G1b PEDV available in our lab, the full-length of the G1b PEDV S protein derived from a historic Taiwan strain was generated and used as an antigen to immunize pigs for the generation of hyperimmune serum against S protein from G1b PEDV strains. The S protein, in particular S1, has been demonstrated to have a relatively high heterogeneity between genotypes and clusters (the identity of amino acid ranges from 91 to 99.6%) compared with other proteins [43]. The amino acid sequence of full-length S and S^1–501^ of the G2b PEDV-PT strain share approximately 94% and 86–88% identity with G1b PEDV S proteins, respectively. We demonstrated that the S^1–501^ ELISAs derived from the G2b PEDV-PT strain, showed a comparable performance with the full-length S-based ELISA in detecting homologous G2b and heterologous G1b PEDV-specific antibodies.

## Conclusion

In this study, both full-length S-based and S^1–501^-based ELISAs performed well in tests with serum samples from different sources as well as with colostrum samples from sow. Moreover, the two ELISAs were suitable for use in the detection of both anti-G1b and G2b PEDV antibodies. Taking into account its performance and cost, S^1–501^-based ELISA, with a sensitivity and specificity of close to 100% and a high protein yield, is the best choice for developing a PEDV ELISA.

## Methods

### Ethics statement

The procedures involving animals were approved and permitted by the Institutional Animal Care and Use Committee (IACUC) of National Taiwan University (NTU; Taiwan, Republic of China) and carried out under the regulations of the IACUC protocol at NTU with the approval number of 106-EL-00054.

### Specimen information

A total of 303 serum samples were used in this study: 213 negative control samples from high managed, no PEDV exposure history farms (199 samples from the conventional farm and 14 samples from an SPF farm); and 90 positive control samples from pigs with PEDV-G2b infection history (50 samples from G2b PEDV-PT challenged conventional post-weaning pigs, 14 samples from G2b PEDV-PT challenged SPF pigs, and 26 samples from sows received G2b PEDV strain feedback). Hence, the serum samples from pigs used in this study can be divided in to five groups: conventional PEDV naïve (C-N) pigs; experimental G2b PEDV-PT inoculated conventional (C-I) pigs; specific pathogen-free PEDV naïve (SPF-N) pigs; experimental G2b PEDV-PT inoculated SPF (SPF-I) pigs; and G2b PEDV-feedback sows (Sow-F) (Table 1). All PEDV-PT experimentally challenged animals orally received 5 × 10^5^ TCID_50_ of G2b PEDV-PT passage 6&7. The serum samples from these animals were collected two weeks after the PEDV challenge. All post-weaning SPF pigs were purchased from the Animal Technology Institute of Taiwan (ATIT). For detection of PEDV specific colostrum IgA, twenty-three colostrum samples from PEDV sero-positive conventional sows with PED outbreak and feedback history in the previous 2 months and 5 colostrum samples from PEDV sero-negative SPF sows were used.

### PEDV isolation and PEDV-infected Vero cell preparation

The G2b PEDV-PT strain (GenBank: KY929405) was prepared as previously described [44]. The 6th passage of PEDV-PT (PEDV-PT-P6) was used as the antigen for the immunostainings in the present study. In brief, to prepare the virus-infected Vero cell plates with visible cytopathic effect (CPE), i.e. the presence of syncytial cells, the Vero cells were seeded onto 96-well culture plates (Thermo Fisher Scientific, Waltham, MA, USA) with a 90% confluence after 18 h. The cells were washed with PBS (Gibco, Gaithersburg, MD, USA) three times and inoculated with 1000 TCID_50_/mL of PEDV-PT-P6 diluted in post-inoculation (PI) medium, containing DMEM (Gibco), 0.3% tryptose phosphate broth (Sigma, St. Louis, MO, USA), 0.02% yeast extract (Acumedia, Lansing, CA, USA), and 10 μg/mL of trypsin (Gibco). After 24 h of incubation, the CPE was observed, the supernatant was discarded and the PEDV-infected Vero cells with 10% area covered by cytopathic effect (CPE) were fixed with 80% acetone (Sigma) for 20 min. After removing the acetone and air-drying for 1 h, the plates were stored at − 20 °C until use.

### Immunostainings of pig serum and colostrum against PEDV-infected Vero cells

To characterize and determine the PEDV-specific antibody titers of the serum and colostrum samples used in this study, the immunostaining of PEDV-infected Vero cells (ICC and IFA) was used as the gold standard. To determine the immunostaining titers of each samples, the serum or the colostrum was serially diluted with PBS (Gibco) from 160-fold to 20,480-fold in a volume of 200 μL/well. The samples were added to the plates of PEDV-infected Vero cells prepared previously, incubated for 1 h at room temperature with gentle shaking, and washed six times with PBS (Gibco). Next, 200 μL of the goat-anti-swine IgG secondary antibody conjugated with horseradish peroxidase (HRP) (KPL, SeraCare, MA, USA) or conjugated with fluorescence (Jackson Laboratory, ME, USA) was diluted 200-fold and added to the wells and incubated for 1 h. After washing six times with PBS (Gibco), the staining was visualized using the EnVision-DAB+ system (Dako, Santa Clara, CA, USA) or by observing the fluorescence under a microscope. To standardize the coloration procedure, each plate included the same positive serum diluted 5120-fold as the positive control and the color development was stopped once the positive control colorized. The color development was evaluated using an inverted light/fluorescence microscope. To avoid any possible non-specific signals of the immunostaining, a signal was only considered positive when appearing on syncytial Vero cells, which show the CPE. If the signals appeared on the morphologically normal Vero cells but not on the CPE cells, the result was considered negative.

### Protein expression and characterization of different lengths of S protein of G2b PEDV-PT

The full-length and truncated S proteins of G2b PEDV-PT, including protein sequences containing aa 1–435(S^1–435^), 1–485 (S^1–485^), 1–501 (S^1–501^), 1–509 (S^1–509^), 1–575 (S^1–575^), and 1–639 (S^1–639^), were constructed and expressed in HEK-293 cells as previously described [17, 32]. These PEDV S-expressing HEK-293 cells were selected using geneticin (Gibco) containing DMEM (Gibco) after two weeks and became stable protein expressing cell lines. To compare the protein yield of the full-length and truncated S proteins of G2b PEDV-PT expressing HEK cells, each protein was purified from 40 mL FreeStyle 293 expression medium (Gibco) and eluted with 1 mL buffer as previously described [17, 32]. The concentration of each S protein was measured using the Pierce™ BCA Protein Assay Kit (Thermo Fisher Scientific) according to the manufacturer’s protocol. To determine the purity and quality of the truncated S protein used for ELISA, the truncated S protein was first denatured in NuPAGE® LDS sample buffer (Thermo Fisher Scientific) containing the NuPAGE® reducing agent (Thermo Fisher Scientific), and boiled at 95 °C for 5 min. The denatured protein was separated by 10% SDS-PAGE and detected by the Coomassie blue (Bio-rad) protein dye.

### Commercial PEDV enzyme-linked immunosorbent assays (ELISAs)

A commercial N protein-based PEDV ELISA kit, ID Screen® PEDV Indirect (IDVet, Montpellier, France), was used in this study according to the manufacturer’s instructions. Briefly, 10 μL of serum was added to each well containing 90 μL of dilution buffer 11 (IDVet), resulting in a ten-fold dilution. After incubation at 25 °C for 45 min, the wells were washed three times with Wash solution (IDVet), then 100 μL of 1× conjugate diluted in dilution buffer 3 (IDVet) was added. This mixture was incubated for another 30 min. After washing three times, 100 μL of substrate (IDVet) was added to the wells and allowed to react for coloration for 15 min before the stop solution (IDVet) was used to stop the coloration reaction. The plates were read at 405 nm using the EMax Plus Microplate Reader (Molecular Devices, Crawley, UK). As per the manufacturer’s instructions, the S/P ratio was interpreted as negative when the S/P ratio < 0.6 and positive when the S/P ratio ≥ 0.6.

### Development of full-length S-based and S^1–501^-based ELISAs

The recombinant S proteins were diluted to 2 μg/mL with coating buffer (KPL, SeraCare, MA, USA) and were coated on a Nunc maxi-soap plate (Thermo Fisher Scientific) at 4 °C for 16 h. After washing six times with washing buffer (KPL, SeraCare), each well was blocked with 300 μL blocking buffer (KPL, SeraCare) at room temperature for 1 h. The serum samples were diluted 40-fold in blocking buffer and then 100 μL was added per well to washed plates and incubated for 1 h at room temperature. After washing six times, as previously described, HRP-conjugated goat-anti-swine IgG antibody (KPL, SeraCare) was added to the wells at 1:1000 dilution and the plates were then incubated for 1 h at room temperature. The coloration procedure was initiated by applying 50 μL/well of ABTS® Peroxidase Substrate System (KPL, SeraCare) onto the washed plates, and the coloration step was stopped by adding 50 μL/well stopping solution (KPL, SeraCare). The signals were read at 405 nm using the EMax Plus Microplate Reader (Molecular Devices). In the ELISA assays, a double-positive sample for both immunostaining against PEDV-infected Vero cells and commercial PEDV ELISA was chosen as the inter-experimental positive control; similarly, a double-negative serum was chosen as the negative control in this study.

### Determination of cut-off value, sensitivity, and specificity of ELISA

To determine the cut-off value of the different truncated S-based ELISAs, 213 negative serum samples (from the C-N and SPF-N groups) and 90 positive serum samples (from the C-I, SPF-I, and Sow-F groups), which were used for the immunostaining against PEDV-infected Vero cells, were analyzed in duplicate by these ELISAs and data are expressed as the S/P ratio. The optimal cut-off value for each ELISA was analyzed and determined by ROC analysis and SPSS Statistics to obtain the best combination of sensitivity and specificity.

### Statistical measurement of testing agreement

To measure the inter-rater agreements of different ELISAs, the Cohen’s kappa coefficient values between the results of immunostaining, commercial PEDV-N ELISA, full-length S-based ELISA, and S^1–501^-based ELISA were calculated and compared. The strength of agreements was considered as described: kappa value ≦ 0: poor; 0.01–0.20: slight; 0.21–0.40: fair; 0.41–0.60: moderate; 0.61–0.80: substantial; 0.81–1: almost perfect. The repeatability of the full-length S-based ELISA and S^1–501^-based ELISA was evaluated by analyzing all of the serum samples in duplicate and obtaining the mean S/P ratio of each sample.

### Colostrum IgA detection using full-length S-based and S^1–501^-based ELISAs

To evaluate whether the S-based ELISAs developed in the present study could detect PEDV specific mucosal IgA, colostrum from PEDV seropositive and negative pigs was used. Five colostrum samples from the PEDV sero-negative SPF sows and 23 colostrum samples from the PEDV sero-positive conventional sows pre-exposed with G2b PEDV by natural infection or feedback were included in this study. The colostrum was centrifuged at 3000 rpm for 30 min to obtain the milk serum. The ELISA procedure as described above was followed with only two modifications: The milk serum was diluted 80-fold and used as the primary antibody and the anti-pig IgA HRP-conjugated antibody (Sigma) was diluted 10,000-fold and used as the secondary antibody. The readout of the ELISA was calculated as the S/P ratio.

### Construction, expression, and purification of G1b PEDV S protein

To determine the cross-reactivity of our G2b PEDV-PT S-based ELISA with a G1b PEDV, it was first necessary to produce a G1b PEDV S protein in order to obtain the porcine G1b PEDV hyperimmune serum. The G1b PEDV used in this study was Taiwan historic PEDV strain (GenBank: HC070225). The gene encoding the S protein was synthesized, codon-optimized, and cloned into a pcDNA™ 3.1/V5-His TOPO® vector (Invitrogen), as previously described [17, 32]. The characterization of the S protein of the Taiwan historic PEDV-HC070225 strain was verified using western blotting as previously described [17, 32].

### Evaluation of cross-reactivity of G2b PEDV-PT S-based ELISAs with G1b PEDV

A post-weaning, conventional PEDV naïve (C-N) pig was purchased and injected intramuscularly with 50 μg of Taiwan historic PEDV strain (GenBank: HC070225) S protein with Freund’s adjuvant (Sigma) three times at 2-week intervals. The serum sample was obtained two weeks after the third injection. The serum sample was serially diluted from 160-fold to 20,480-fold and ICC was carried out against G2b-PEDV-PT infected Vero cells to determine the titer of the immunostaining. Finally, the serum was used in both the commercial N protein-based ID Screen® PEDV Indirect (IDVet) ELISA and our G2b PEDV-PT S-based ELISAs, following the same procedure described above, to evaluate the cross-reactivity of the ELISAs.

## Supplementary information

**Additional file 1: Figure S1.** The evaluation of purity of S^1–501^ protein on the sodium dodecyl sulfate (SDS)-polyacrylamide gel electrophoresis (PAGE). The purified recombinant S^1–501^ was separated by the SDS-PAGE and stained with the Coomassie blue protein dye. The molecular weight of the recombinant S^1–501^ protein was approximately 75 kDa, respectively. The protein ladder (M) is shown in kilodalton (kDa). The star icon indicated the S^1–501^ protein on the SDS-PAGE.

## Data Availability

The datasets used and/or analyzed during the current study are available from the corresponding author on reasonable request.

## Abbreviations

ATCC: American Type Culture Collection
C-I: Experimental PEDV inoculated conventional pigs
C-N: Conventional PEDV naïve pigs
COE epitope: CO-26 K equivalent epitope
CPE: Cytopathic effect
E: Envelop
ELISA: Indirect enzyme-linked immunosorbent assay
G2: Genotype 2
HEK 293: Human embryonic cell 293
HRP: Horseradish peroxidase
IACUC: Institutional Animal Care and Use Committee
ICC: Immunocytochemical
M: Membrane
N: Nucleocapsid
NTU: National Taiwan University
PED: Porcine epidemic diarrhea
PEDV: Porcine epidemic diarrhea virus
PEDV-PT: G2b PEDV Pintung 52 strain
PEDV-PT-P6: G2b PEDV Pintung 52 strain passage 6
PI medium: Post-inoculation medium
S: Spike
S/P ratio: Sample to positive ratio
S^1–435^: a.a.1–435 of S protein
S^1–485^: a.a.1–485 of S protein
S^1–501^: a.a.1–501 of S protein
S^1–501^: Amino acid 1–501 of G2b PEDV-PT S protein
S^1–509^: a.a.1–509 of S protein
S^1–575^: a.a.1–575 of S protein
S^1–639^: a.a.1–639 of S protein
SD: Standard deviation
Sow-F: PEDV-feedback sows
SPF-I: Experimental PEDV inoculated SPF pigs
SPF-N: Specific pathogen free PEDV naïve pigs
