# Supplementary figures and images for: Development and comparison of enzyme-linked immunosorbent assays based on recombinant trimeric full-length and truncated spike proteins for detecting antibodies against porcine epidemic diarrhea virus
Source: BMC Vet Res. 2019 Nov 27;15:421. doi: 10.1186/s12917-019-2171-7 (PMC6880432; doi:10.1186/s12917-019-2171-7)

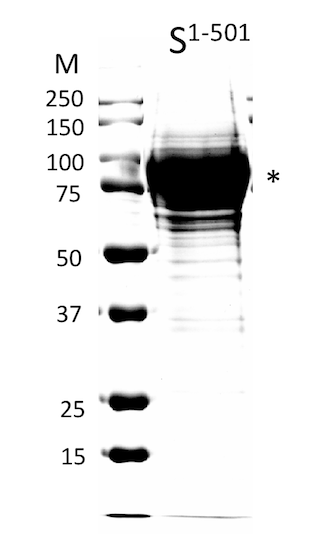

Supplement: Supplementary file 1 — Additional file 1: Figure S1. The evaluation of purity of S1–501 protein on the sodium dodecyl sulfate (SDS)-polyacrylamide gel electrophoresis (PAGE). The purified recombinant S1–501 was separated by the SDS-PAGE and stained with the Coomassie blue protein dye. The molecular weight of the recombinant S1–501 protein was approximately 75 kDa, respectively. The protein ladder (M) is shown in kilodalton (kDa). The star icon indicated the S1–501 protein on the SDS-PAGE. [file 12917_2019_2171_MOESM1_ESM.tiff]
